# Exploration of Development Parameters for IgG Microspheres Using an Automated Platform for High-Throughput Formulation Screening

**DOI:** 10.3390/ph19081203

**Published:** 2026-08-01

**Authors:** Puneet Tyagi, Donyeil Hoy, Corina Badea-Mic, Anders Eckburg, Daniel Contaifer, Max Brodie, Kartik Raman, Kenan Pandza

**Affiliations:** 1 Dosage Form Design and Development, BioPharmaceuticals R&D, AstraZeneca, Gaithersburg, MD 20878, USA; puneet.tyagi@astrazeneca.com (P.T.); donyeil.hoy@astrazeneca.com (D.H.); 2 Persist AI, 1001 Riverside Pkwy, West Sacramento, CA 95605, USA; corina@persist-ai.com (C.B.-M.); anders@persist-ai.com (A.E.); daniel@persist-ai.com (D.C.); max@persist-ai.com (M.B.); kraman@persist-ai.com (K.R.)

**Keywords:** microspheres, PLGA, PLA, loading, sustained release, formulations, IgG

## Abstract

**Background/Objectives:** PLGA/PLA microspheres are attractive candidates for depot delivery of protein therapeutics, but formulation development for antibodies remains challenging because protein encapsulation, burst release, stability, and polymer composition must be balanced across a large design space. This study evaluated an automated high-throughput formulation workflow for preparing and screening IgG-loaded pegylated PLGA/PLA microspheres, with emphasis on loading and early release behavior. **Methods:** Microspheres were prepared using an automated W/O/W emulsion workflow consisting of primary emulsion formation, microfluidic secondary emulsification, solvent removal, washing, and lyophilization. A formulation library was generated by varying polymer chemistry, polymer concentration, polymer blends, IgG concentration, and additives. Formulations were evaluated for IgG loading and in vitro release over the first 7 days using automated sampling and protein quantification. Statistical analyses were used to identify formulation variables associated with loading, burst release, day-7 release, and Weibull-derived kinetic descriptors. **Results:** Across 129 formulations, IgG loading was generally in the low-to-mid single-digit percentage range. Additive class and IgG concentration in the dispersed phase were the formulation variables most strongly associated with early release behavior. Additive-free systems were associated with lower burst release, whereas non-polymeric additives were associated with higher burst and faster early release. Day-7 release differences were less robust after correction for variance heterogeneity and resampling. **Conclusions:** The automated workflow enabled rapid preparation and comparative screening of IgG-loaded PLGA/PLA microspheres. The findings support the use of high-throughput automated screening to identify formulation variables associated with burst modulation and early release behavior. Because release was evaluated over 7 days and only one model IgG was used, the results should be interpreted as an early-stage formulation screening study rather than definitive evidence of long-term antibody depot performance.

## 1. Introduction

Long-acting injectable (LAI) formulations have emerged as a transformative approach in biopharmaceutical delivery, offering improved patient adherence and reduced dosing frequency, particularly for chronic and prophylactic therapies [1,2,3]. By reducing the frequency of administration, LAI products can alleviate treatment burden, enhance compliance, and improve quality of life—attributes especially valuable in indications requiring lifelong management. These benefits also support product differentiation and life-cycle management, making LAI strategies increasingly attractive from both clinical and commercial perspectives [4,5].

While LAI formulations are well established for small molecules and peptides, their application to therapeutic monoclonal antibodies (mAbs) remains limited [6]. Technologies such as poly(lactic-co-glycolic acid) (PLGA)-based microspheres, which are widely used in marketed peptide depots, offer a promising platform due to their biodegradability, tunable release profiles, and regulatory familiarity [7,8,9]. However, developing robust PLGA-based depot systems for large, structurally complex immunoglobulins (IgG) presents distinct challenges. The multifactorial nature of formulation design—encompassing polymer chemistry, excipient interactions, and process conditions—must be carefully balanced with the need to preserve antibody stability, conformation, and function throughout encapsulation and sustained release [10,11,12,13]. These challenges have made long-acting formulations of mAbs an active and unmet area of innovation [14].

These LAI presentations for mAbs are increasingly recognized not only for their potential to enhance pharmacokinetic profiles but also as strategic tools in life-cycle management (LCM). By offering new delivery formats of “soon-to-be-off-patent” mAbs, long-acting formulations allow for market differentiation [15], expanded intellectual property, and sustained commercial value [1]. As competitive landscapes tighten and biosimilar threats emerge, long-acting presentations serve as timely innovations to extend product lifespans while simultaneously meeting patient and healthcare provider preferences for more convenient dosing regimens [16].

Despite their promise, the development of depot systems for complex proteins like IgG remains technically challenging. Encapsulation within biodegradable polymers such as PLGA requires careful optimization to balance release kinetics, stability, and functional integrity of the protein over extended periods [10,11,12,13]. The multidimensional nature of formulation variables—including polymer type, concentration, excipient selection, and process conditions—necessitates a high-throughput, data-rich approach to formulation design.

To address these challenges, we employed a high-throughput platform that integrates automated formulation development and analytical feedback loops to accelerate screening and optimization. This platform allows multiplexed microsphere formulation building and testing via robotic systems, with continuous analytical readouts (e.g., payload loading and release kinetics) enabling efficient exploration of complex formulation landscapes [17].

In this study, we report the development of PLGA/PLA-based microsphere formulations capable of sustained antibody release using human immunoglobulin G (hIgG) as a payload. A total of 129 formulations were screened by varying polymers, blends, concentrations, and additives, and the impact of these parameters was analyzed to identify key factors governing release.

Our findings highlight a scalable strategy for LAI development for protein payloads and provide a formulation framework with both clinical and commercial potential for advancing long-acting antibody therapeutics.

## 2. Results

### 2.1. Formulations and Parameter Selection

A total of 129 microsphere formulations were made using an automated formulation building platform, where the formulation parameters were selectively varied (Table S2 in Supplementary Materials). Formulations were built using W/O/W methods to encapsulate hIgG as a payload. The parameters selected as the variables in the protocol were those anticipated to show an impact on the IgG loading and release [18,19,20,21,22,23,24].

### 2.2. Polymer Chemistry

The chemistry of PLGA/PLA polymers (i.e., inherent viscosity as the factor of polymer molecular weight, terminal cap, and lactide:glycolide (L:G) ratio) is an essential factor that controls the polymer hydrolysis rate and payload release [25,26,27]. To evaluate the influence of polymer chemistry on IgG encapsulation and release, multiple polymers were tested.

Selection criteria included varying L:G ratios and internal viscosity. Selected polymers had high (0.6–0.9) and low (0.3–0.7) inherent viscosity. In addition to PLA polymers, the PLGA polymers had a 50:50 and 75:25 L:G ratio. All polymers had polymer chains terminated with PEG-5000. This modification allows for the formation of a hydrophobic pocket within the microsphere matrix compatible with hydrophilic protein molecules [28,29]. To increase polymer chemistry diversity, a secondary polymer was added to the primary polymer. The polymer mixture was made at different ratios (3:1, 1:1, and 1:3), resulting in a copolymer matrix of blended internal viscosity or L:G ratio (Table S3 in Supplementary Materials).

### 2.3. Polymer Concentration

Polymer concentration can impact both payload loading and release kinetics [27,30]. Polymer was used at two concentrations in the total dispersed phase. Low polymer concentration was used to allow fast protein release. In total, 78 formulations were made with 3.3% drug in the dispersed phase, and 51 formulations were made with 6.7% polymer (Table S3 in Supplementary Materials).

### 2.4. Additives

A broad additive panel, generally divided into polymers and non-polymers, was tested to assess their ability to modulate protein–polymer interactions and alter release kinetics. The following additives that showed compatible solubility in the disperse phase were included in the screening: Alginic acid [31], Dextran sulfate [32], Distearoyl-Phosphocholine [33], Heparin [34], Polyallylamine [35], Arginine [36], Ammonium sulfate [37], Polyethyleneimine [38], Poly-L-Lysine [39], Chitosan [40], Na-Hyaluronate [41], Polysorbate 80 [42], and Polyacrylamide [43]. Additives were incorporated at 0.01–0.05% (*w*/*v*) into either the organic phase or the internal aqueous phase (Table S2 in Supplementary Materials). Formulations were grouped into classes, defined as:

- No-additive: No excipients added beyond polymer and drug; baseline control condition.
- Polymeric additives: High molecular weight excipients (e.g., hydrophilic polymers) expected to modulate porosity or hydration.
- Non-polymeric additives: Small-molecule or surfactant additives expected to alter interfacial stability or diffusion.

### 2.5. Protein Concentration

Protein concentration can have an impact on the loading but also on the diffusion rate in the early stage of the release [22]. hIgG was used at 1.25, 2.0, 3.75, and 10 mg/mL total concentration in the dispersed phase (Table S2 in Supplementary Materials). To prevent protein denaturation by exposure to solvent, the hIgG was prepared as the aqueous phase of the primary emulsion. Poloxamer 188 was added to create the interphase and prevent the interaction of the protein with the solvent in the organic phase [20].

### 2.6. Formulation Testing

The formulations were evaluated for drug loading using a dissolution assay, and the released IgG was evaluated using a BCA assay (Table 1).

Subsequently, the release profiles in formulations MA31-MA129 were evaluated using an automated release testing platform. Formulations with resulting 0% drug loading were not assessed for release.

**Figure 1:**
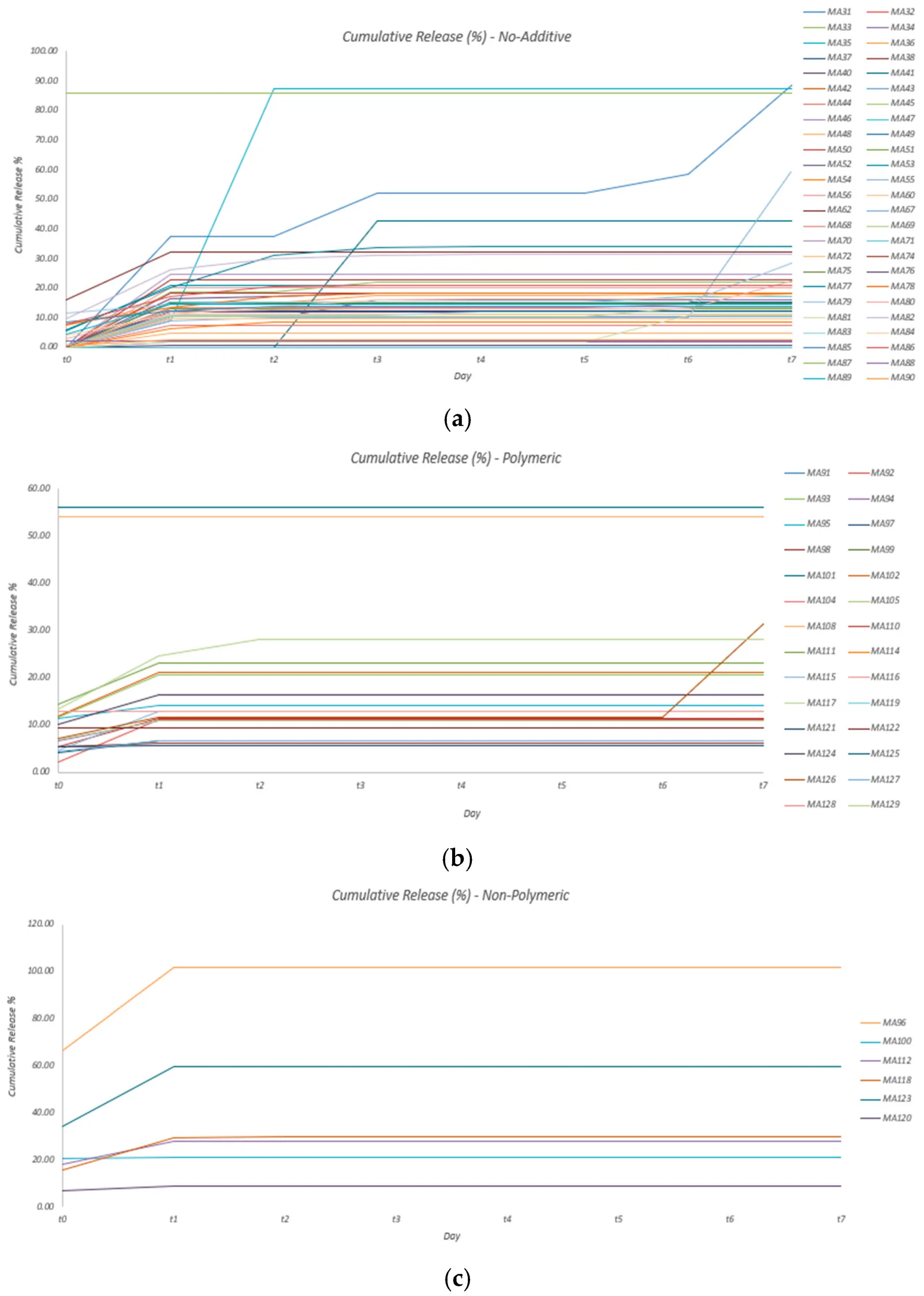
Cumulative release of IgG over time as the percentage of the total IgG in microspheres. Release is shown for formulations with no additives. (**a**), formulations with polymeric additives (**b**), and formulations with non-polymeric additives (**c**).

Briefly, the microspheres are incubated in the release buffer (PBS, pH 7.4). The release buffer is collected and replenished with fresh media at daily intervals for 7 days. Released IgG was measured in the release buffer with the BCA assay. The release is expressed as a percentage of the total IgG released from the microspheres at a given time. The release kinetics are analyzed as a cumulative release trend over time (Figure 1a–c). Rather than analyzing the complete release profile over the lifetime of the microsphere, the focus of this study was to evaluate the parameter’s impact at the initial release period (i.e., 7 days) as a rapid test for formulation screening.

### 2.7. Drug Loading Outcomes

**Figure 2:**
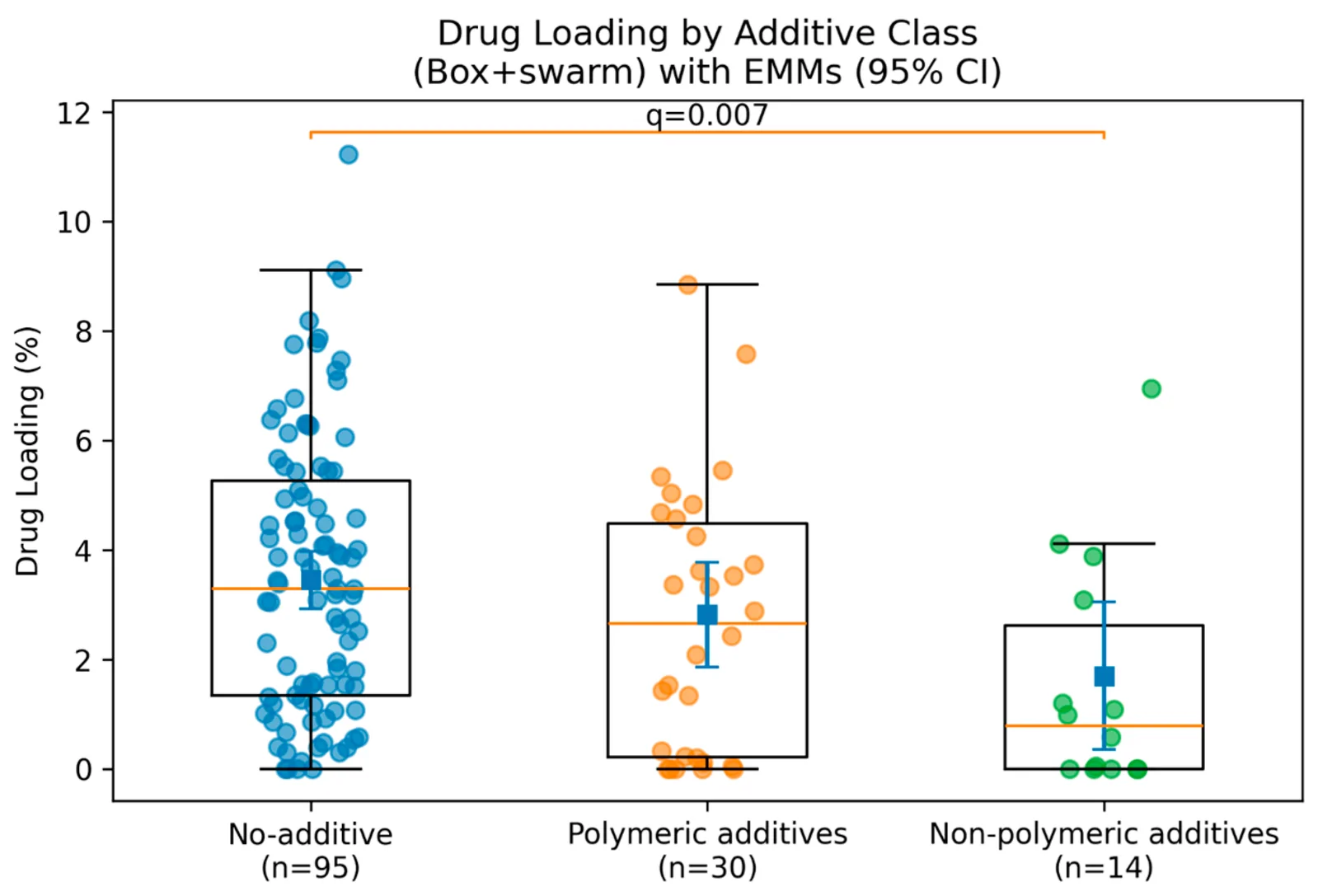
Drug Loading (%) by additive class. Boxplots with jittered points (n per class shown). Squares and whiskers show EMMs (±95% CI) from ANCOVA with HC3 errors adjusted for covariates. Omnibus ANCOVA: F = 2.84, *p* = 0.062, partial η^2^ = 0.041. Robustness checks: HC3 Wald *p* = 0.096; Freedman–Lane permutation *p* = 0.466. FDR-adjusted Dunn test significant for No-additive vs. Non-polymeric (q_FDR_ = 0.0066).

Drug loading expressed as the *w*/*w* percentage of the protein in microspheres showed modest variability across additive classes. ANCOVA with HC3-robust errors suggested a trend-level effect (F = 2.84, *p* = 0.062, partial η^2^ = 0.041), which was not supported under permutation testing (*p* = 0.466). Despite the non-significant omnibus test, unadjusted Dunn post hoc contrasts with FDR control indicated a significant difference between No-additive and Non-polymeric formulations (q_FDR_ = 0.007), with No-additive systems showing higher loading (Figure 2). EMMs confirmed the ordering No-additive ≥ Polymeric > Non-polymeric, but other pairwise comparisons were not significant.

**Figure 3:**
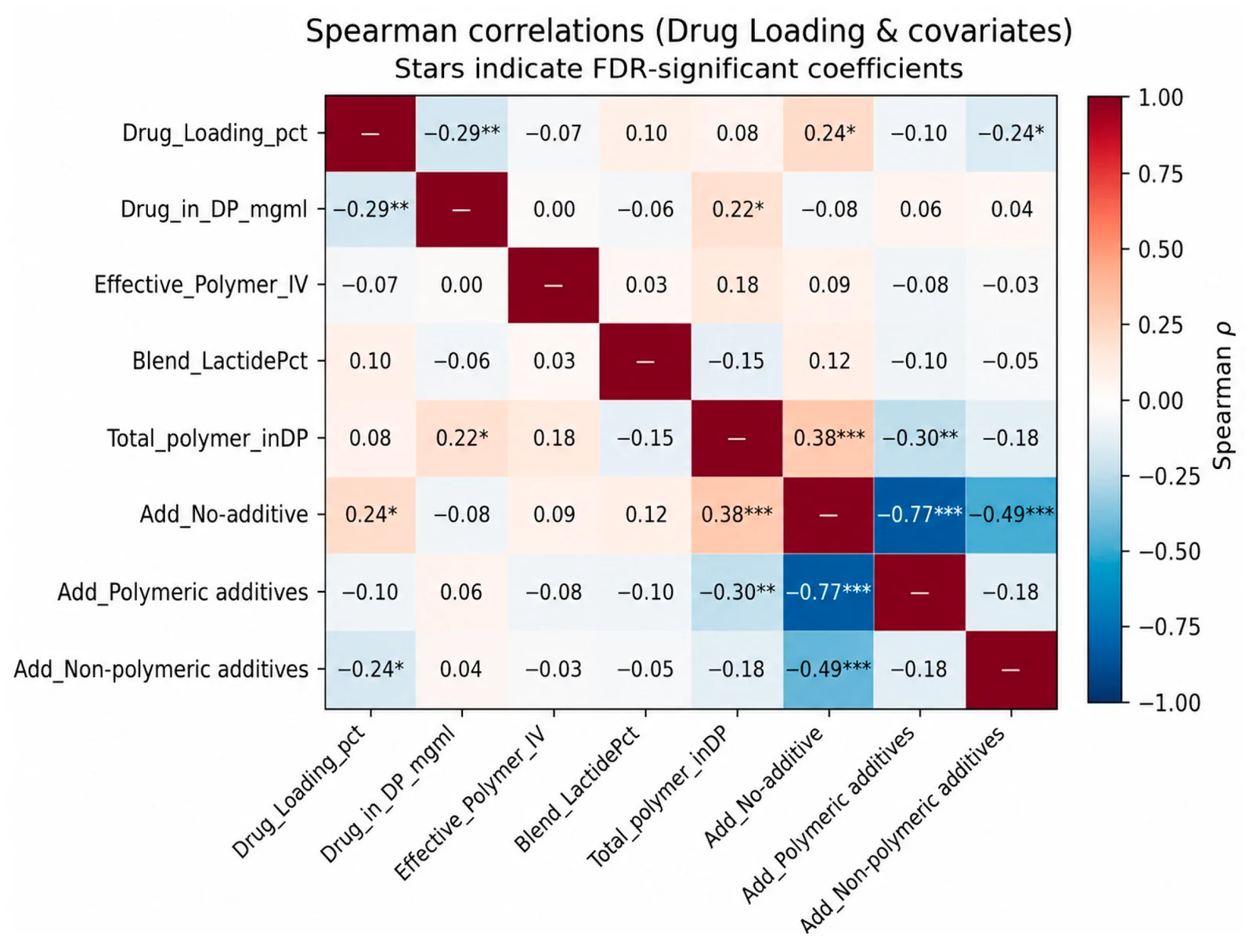
Spearman correlation matrix for Drug Loading (%) and formulation variables. Heatmap shows correlation coefficients (ρ; red = positive, blue = negative, white ≈ 0). Significant associations after FDR: dispersed-phase drug concentration (ρ ≈ −0.29, q_FDR_ < 0.01), Non-polymeric additives (ρ ≈ −0.24, q_FDR_ < 0.05), and No-additive formulations (ρ ≈ +0.24, q_FDR_ < 0.05). Stars denote FDR significance (q_FDR_: * < 0.05, ** < 0.01, *** < 0.001).

In contrast, the Spearman correlation analysis provided complementary evidence for parameter effects on loading (Figure 3). Drug loading was negatively associated with dispersed-phase drug concentration (ρ ≈ −0.29, q_FDR_ < 0.01) and Non-polymeric additives (ρ ≈ −0.24, q_FDR_ < 0.05), and positively associated with No-additive formulations (ρ ≈ +0.24, q_FDR_ < 0.05). These loading-level associations were weak and, for additive class, not robust under resampling, and should therefore be regarded as exploratory and hypothesis generating; the most consistent signal was the negative association with dispersed-phase drug concentration.

### 2.8. Release Outcomes

**Figure 4:**
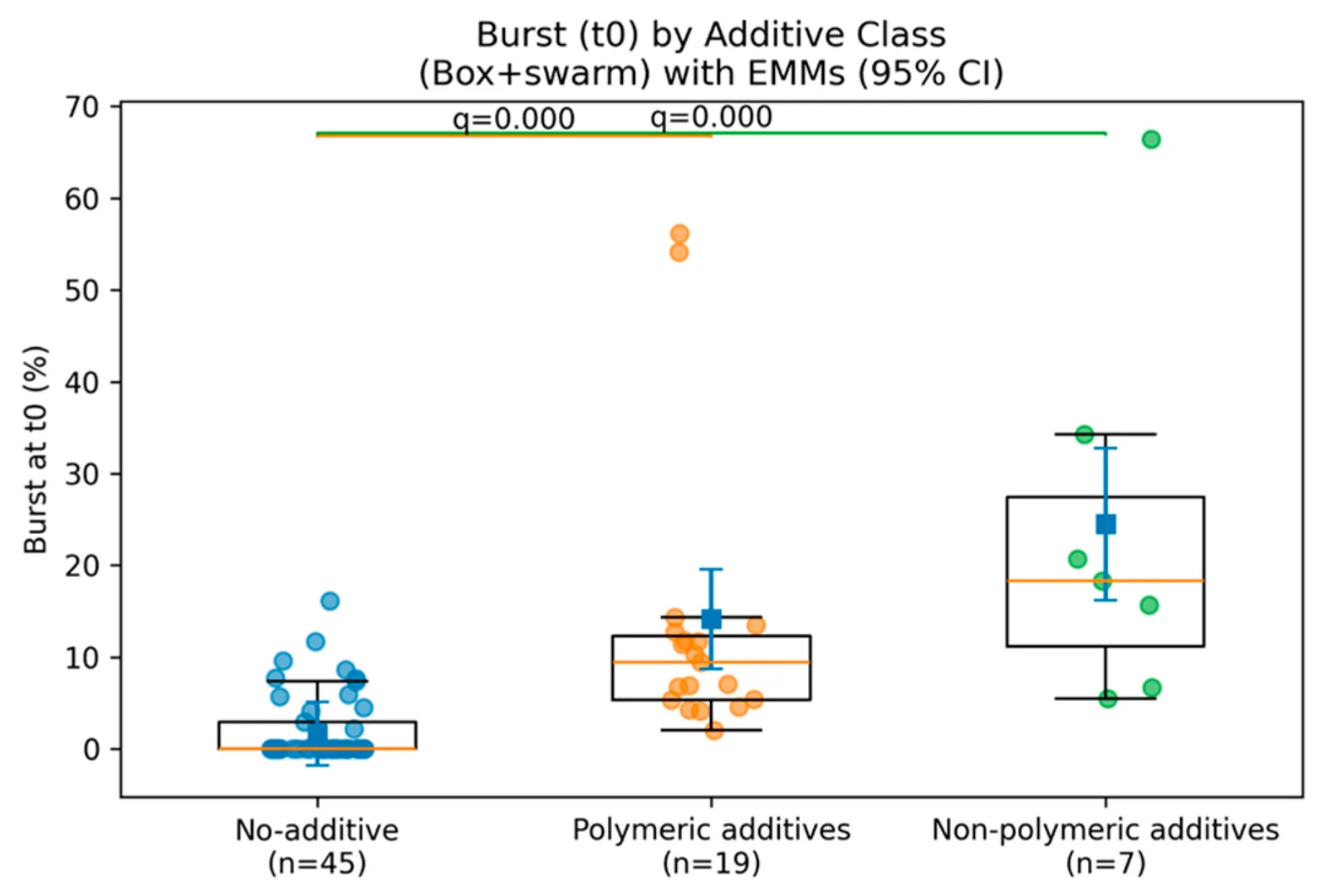
Burst release (t0) by additive class. Boxplots with jittered points (No-additive *n* = 46, Polymeric *n* = 19, Non-polymeric *n* = 7). Squares show EMMs ±95% CI from ANCOVA with HC3 errors. Omnibus ANCOVA: F = 13.60, *p* = 1.2 × 10^−5^, partial η^2^ = 0.298. Robustness checks: HC3 block *p* = 0.0018; permutation *p* = 0.0024. EMMs: No-additive 1.65% (−1.79–5.09), Polymeric 14.18% (8.75–19.61), Non-polymeric 24.50% (16.22–32.79).

**Figure 5:**
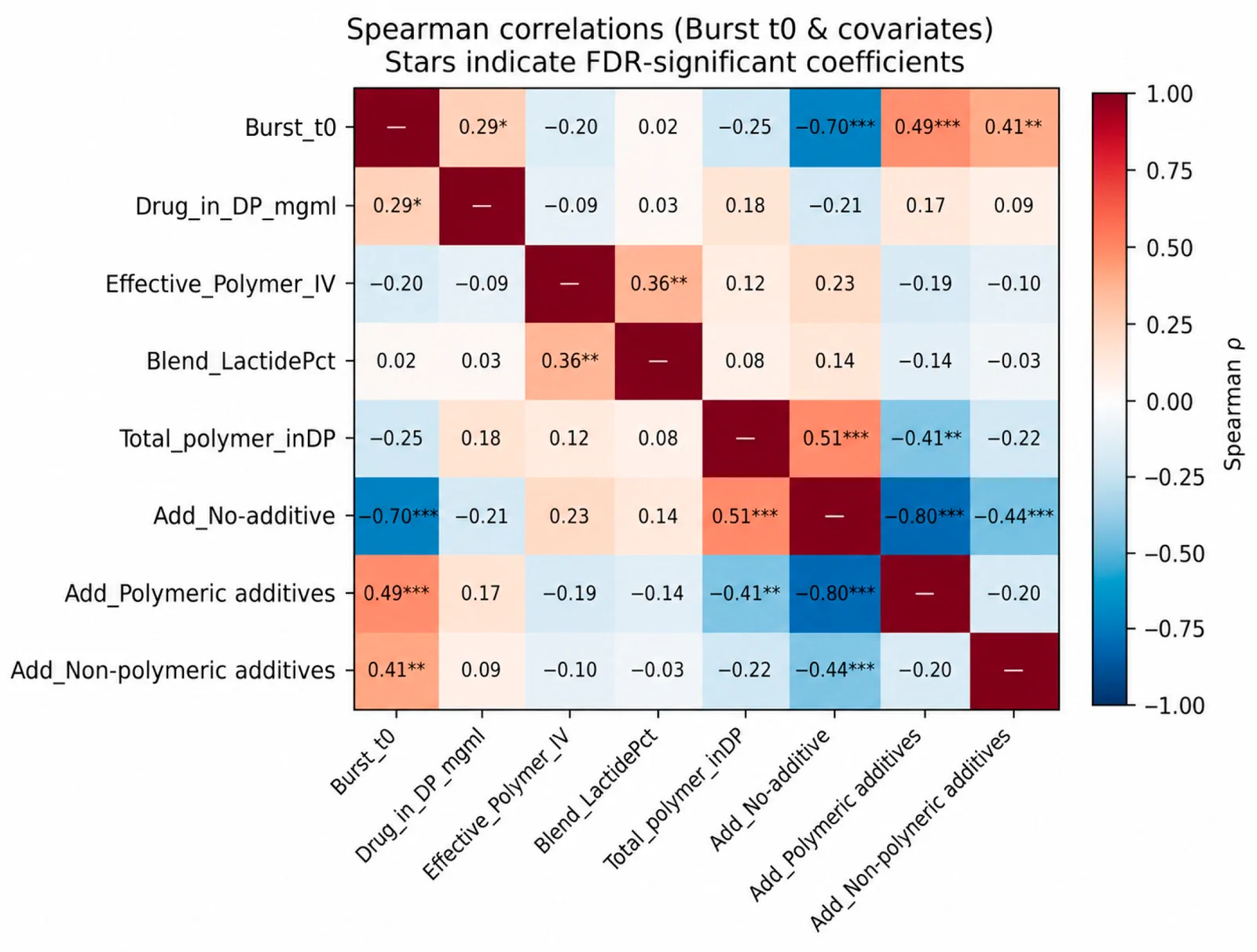
Spearman correlation matrix for burst release (t0) and formulation variables. Heatmap shows correlation coefficients (ρ; red = positive, blue = negative, white ≈ 0). Significant associations after FDR: No-additive (ρ ≈ −0.64, q_FDR_ < 0.001), Polymeric additives (ρ ≈ +0.46, q_FDR_ < 0.001), Non-polymeric additives (ρ ≈ +0.35, q_FDR_ < 0.05), and dispersed-phase drug concentration (ρ ≈ +0.33, q_FDR_ < 0.01). Stars indicate FDR significance (q_FDR_: * < 0.05, ** < 0.01, *** < 0.001).

The additive class exerted strong and reliable effects on release. Burst release (t0) showed the clearest pattern, with No-additive systems minimizing burst, Non-polymeric additives producing the highest burst, and Polymeric additives occupying an intermediate position (Figure 4). Correlation analysis corroborated these associations (Figure 5).

Day-7 cumulative release differed at the nominal parametric level (ANCOVA *p* = 0.0245) but not under heteroskedasticity-consistent correction (p_HC3_ = 0.209) or the Freedman–Lane permutation test (p_perm_ = 0.248); the parametric significance reflects variance heterogeneity arising from the small, more variable Non-polymeric class (*n* = 7), so this difference is treated as nominal and non-robust, with overlapping confidence intervals across classes. Kinetic modeling clarified the underlying behavior. The two Weibull parameters describe distinct aspects of release: β captures the shape of the cumulative curve (the transport/relaxation regime), whereas log τ captures its characteristic timescale (the rate). β did not differ significantly among additive classes, whereas log τ differed strongly and robustly. The natural reading is that the underlying release mechanism is conserved across all three additive classes, and that additives act on the kinetics—accelerating or decelerating release—rather than switching the transport regime (for example, from a diffusion-dominated to an erosion-dominated process). Among the classes, No-additive systems released most slowly, Non-polymeric additives accelerated release, and Polymeric additives were intermediate. Table 2 summarizes these results.

### 2.9. Determinants of Loading and Release

Building on the outcome-level results, predictor-level analyses confirmed that additive class and drug concentration in the dispersed phase were the formulation factors most strongly and reproducibly associated with release behavior. The additive class consistently explained the largest share of variance in both burst and kinetic pacing, with partial η^2^ values exceeding the conventional threshold for large effects. This influence was robust across ANCOVA models and correlation analyses, indicating that the choice of additive is most strongly associated with whether systems release the drug abruptly, steadily, or in a balanced intermediate fashion.

Drug concentration in the dispersed phase emerged as the most reliable continuous covariate. Higher concentrations were consistently linked to reduced day-7 release, likely reflecting a combination of diffusional hindrance and decreased encapsulation efficiency at elevated loads. Importantly, this effect was independent of additive class, suggesting that drug input levels modulate release magnitude even within otherwise well-optimized formulations.

In contrast, the lactide fraction played a more conditional role. While not significant across the dataset, it showed a consistent trend toward slowing kinetics in No-additive systems, suggesting that higher lactide content may partially compensate for the absence of release-modifying excipients. This interaction highlights the potential for polymer composition to serve as a secondary lever when excipient options are limited.

Finally, polymer intrinsic viscosity and total polymer concentration in the dispersed phase contributed negligibly once additive class and drug concentration were accounted for. Their low effect sizes indicate that, within the ranges tested, these parameters provide little independent explanatory power for drug loading or release outcomes.

**Figure 6:**
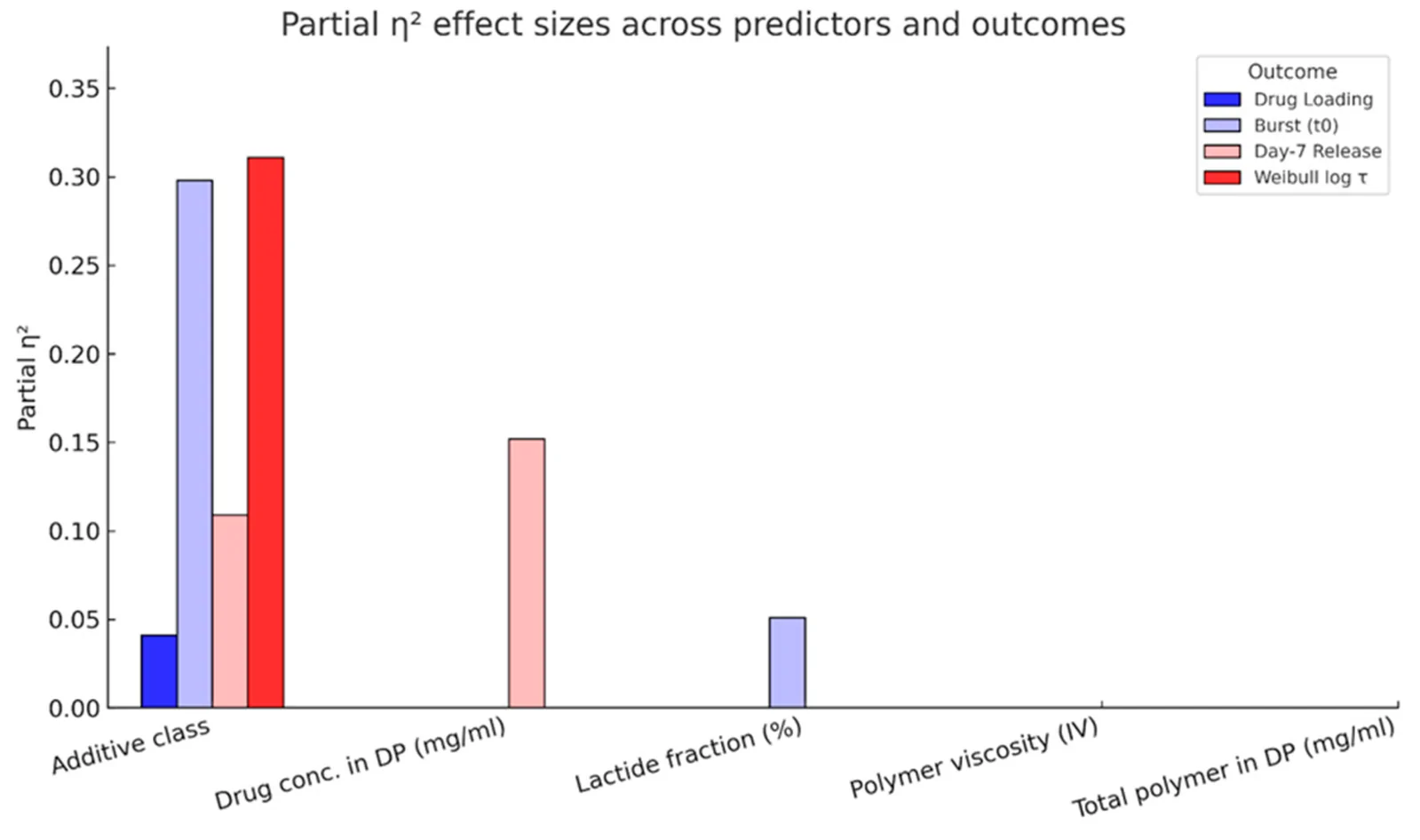
Effect size profiles of predictors. Bar plot highlights the dominant influence of additive class on burst and release kinetics, the strong role of drug concentration in day-7 release, and the minimal contributions of lactide fraction and polymer viscosity.

These relationships are summarized in Table 3, which contrasts the relative influence of predictors across outcomes, and are visualized in Figure 6, where the large effect sizes for additive class and drug concentration stand in clear contrast to the minimal or context-specific roles of other formulation parameters. Together, these findings establish a hierarchy of influences: excipients and drug concentration dominate early release, while polymer composition exerts only modest, situational effects.

To facilitate interpretation of the statistical evidence across outcomes, Table 4 summarizes the concordance between the parametric ANCOVA, HC3-robust inference, and permutation testing. Burst release and Weibull log τ were consistently supported by all three approaches and were therefore classified as robust findings. In contrast, the apparent effect on day-7 release was significant only in the conventional ANCOVA and was classified as nominal, whereas drug loading remained exploratory because evidence arose primarily from the correlation analysis rather than robust model-based inference.

## 3. Discussion

The present study evaluated formulation parameters influencing the encapsulation efficiency and release kinetics, in PLGA/PLA-based microspheres prepared by a double emulsion (W/O/W) method. The overarching goal was to identify conditions that modulate IgG release during the early screening phase.

Taken together, the results suggest that additive choice and drug concentration should be prioritized as the main levers for formulation design, with lactide fraction providing secondary, context-specific modulation and polymer viscosity playing only a minor role. The present analysis indicates that additive class and drug concentration in the dispersed phase are the formulation factors most strongly and reproducibly associated with antibody release from PLGA microspheres (Table 3 and Table 4, Figure 6). The additive class exerted large, robust effects on burst release, while drug concentration showed a strong, consistent influence on day-7 release. In contrast, lactide fraction contributed only in a context-specific manner, and polymer intrinsic viscosity and total polymer concentration were negligible. Together, these results highlight the hierarchy of factors, with additives and drug concentration dominating early release behavior.

Although ANCOVA did not confirm additive class as a significant determinant of drug loading, the correlation structure suggested parameter-dependent modulation. Specifically, loading efficiency decreased with higher dispersed-phase drug concentrations and with non-polymeric additives, while additive-free systems tended to favor higher loading. This complementary evidence indicates that, even if loading effects were not robust in model-based inference, formulation choices can still shape encapsulation efficiency in predictable ways.

The strength and robustness of additive effects across release outcomes underscore their central role in shaping kinetics. No-additive systems consistently minimize burst but also slow release. Non-polymeric additives accelerated release at the expense of an elevated burst. Polymeric additives produced intermediate profiles. These patterns were confirmed by both model-based inference and correlation analyses, reinforcing confidence in their reproducibility. Drug concentration acted in a complementary but distinct way, reliably reducing day-7 release at higher input levels, likely reflecting diffusional constraints or reduced encapsulation efficiency.

From a formulation design perspective, these findings provide practical guidance for tuning early release behavior. Additive choice establishes the overall release framework, while drug concentration modulates its intensity. Lactide fraction may serve as a secondary lever in additive-free systems, but other polymer characteristics, such as intrinsic viscosity, contribute little within the tested ranges. These findings provide evidence that the additive class and drug concentration are the primary factors for tuning burst and early antibody release.

This study should be interpreted as an early-stage, high-throughput formulation screening study rather than a definitive demonstration of long-term antibody depot performance. The 7-day release window was selected to enable rapid comparison of a large formulation library and to identify variables associated with burst release and early release kinetics. This time frame is useful for prioritizing formulations, but it does not establish release over the multi-week or monthly periods typically expected for long-acting injectable products. Longer release studies using selected lead formulations will be required to confirm whether the early release trends observed here translate into extended depot performance.

The release behavior observed in this study is likely governed by multiple coupled processes, including initial diffusion of surface-associated or weakly entrapped IgG, hydration of the polymer matrix, formation of aqueous pores or channels, and later polymer relaxation or erosion. Because particle morphology, porosity, and size distribution were not directly measured in the present screen, mechanistic interpretation must remain cautious. The statistical associations identified here should therefore be viewed as formulation-level trends rather than direct evidence of a single release mechanism.

The use of additive classes provided sufficient group sizes for statistical analysis, but it may obscure differences among individual additives. Therefore, class-level effects should be interpreted as screening-level associations. Additives such as polyethyleneimine, polyallylamine, and ammonium sulfate may be useful as mechanistic probes during early formulation screening, but not all additives evaluated here would necessarily be appropriate for clinical translation due to potential safety or biological effects. Future optimization should prioritize additives with established pharmaceutical acceptability and should evaluate biocompatibility, protein stability, and long-term release for selected lead formulations.

Some limitations should be considered when interpreting these results. First, this was an exploratory study based on a relatively small and imbalanced dataset, which reduced statistical power and may have amplified class-specific variability. Although robust statistical methods were applied, some nominal findings (e.g., drug loading and day-7 release) did not remain significant under resampling. Second, the study focused only on early release (up to 7 days), and longer-term kinetics were not captured. The study used one model IgG and a specific automated W/O/W fabrication workflow. Second, formulations with very low loading may have limited translational relevance; these formulations are useful for identifying unfavorable formulation conditions but should be interpreted cautiously in release modeling. Finally, other potentially relevant formulation factors, such as polymer end-group chemistry or process conditions, were not included. These limitations support the use of the present dataset for hypothesis generation and formulation prioritization, rather than broad generalization across all antibody-loaded PLGA systems.

This work represents the initial phase of a broader, ongoing initiative aimed at enabling sustained delivery of pipeline monoclonal antibodies using long-acting injectable platforms. Building upon the formulation principles and high-throughput screening framework established here, future studies will focus on applying this approach to multiple therapeutic mAbs. In vivo pharmacokinetic and pharmacodynamic studies should be used to evaluate depot performance, bioavailability, and immunogenicity in relevant animal models. These investigations will inform candidate selection, dose regimen design, and device compatibility, ultimately supporting the translation of long-acting formulations into clinical development. This foundational study not only demonstrates technical feasibility but also sets the stage for a systematic evaluation of long-acting antibody delivery as a key enabler for next-generation biologic therapeutics.

## 4. Materials and Methods

### 4.1. Materials

Human immunoglobulin G (hIgG) fraction from human plasma was obtained from Goden West Biologicals (Temecula, CA, USA). PEG–PLGA and PEG-PLA polymers were sourced from Seqens (Ecully, France). Solvent and other excipients were obtained from different suppliers (see Table S1 in Supplementary Materials). All sourced reagents were of analytical grade.

### 4.2. Preparation of Microsphere Formulations

**Figure 7:**
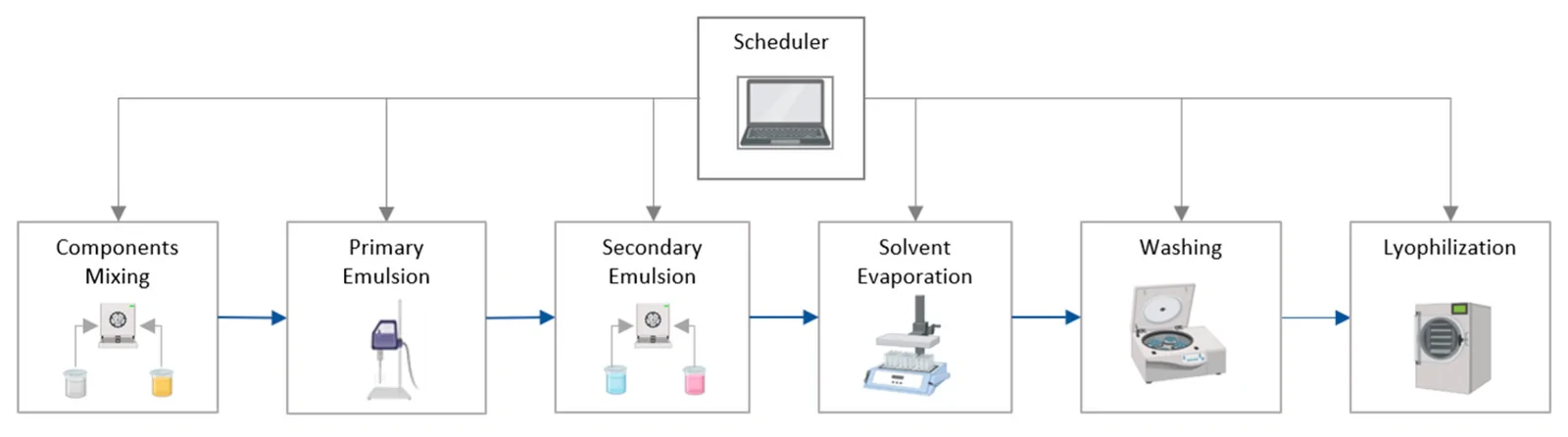
Microsphere preparation workflow with process steps on the automated build platform.

Microspheres were prepared using an automated platform for high-throughput formulation development (Figure 7).

In the first step, the aqueous hIgG solution was prepared by combining hIgG with PBS pH 7.4, followed by the addition of the additive. Additives were prepared in water or DCM prior to addition to the aqueous phase. Phase separation was controlled by adding 0.17% Poloxamer 188 as a secondary additive.

In the second step, the primary emulsion was produced by sonicating the aqueous hIgG solution into an organic polymer solution containing the pegylated PLGA/PLA polymer dissolved in DCM. Sonication conditions and environmental temperature were controlled to reduce protein denaturation prior to encapsulation. Sonication was performed using the Fisherbrand FB705 Sonic Dismembrator (Fisher Scientific, Pittsburgh, PA, USA) with a frequency of 20 kHz, power of 700 W, probe diameter of ½ in (13 mm), and controlled amplitude of 10% (% of maximum) to generate a stable dispersed phase. The emulsion temperature was kept low by incubating on ice during sonication.

In the subsequent step, the secondary emulsion was formed using a microfluidic module that combined the dispersed phase at a rate of 1 mL/min with an aqueous continuous phase (containing 2% PVA) at a rate of 15 mL/min via an array of 20 um diameter precision micropores (20 um diameter) (Micropore, Redcar, UK).

The resulting microdroplets were subjected to solvent evaporation under low pressure (230 mBar) and controlled temperature (25 °C) to remove DCM with Syncore evaporator system (Buchi, New Castle, DE, USA), during which the droplets hardened into microparticles. Microspheres were then washed with water to remove unincorporated protein, polymer residues, and PVA. The washing step was repeated five times to ensure effective removal of unincorporated material. Each time, the microspheres were recovered by centrifugation for 5 min at 1500 rcf. Washed microspheres were finally lyophilized using Lyovapor (Buchi, New Castle, DE, USA) for up to 24 h and stored at −20 °C until further analysis. The process workflow is controlled by centralized schedule control software.

### 4.3. Determination of Loading Efficiency

Loading efficiency was measured by dissolving a known mass of microspheres in DMSO to ensure complete polymer dissolution and release of the encapsulated protein. Samples were vortexed and incubated until fully dissolved. Released protein content was quantified using the bicinchoninic acid (BCA) assay (Sigma-Aldrich, St. Louis, MO, USA) according to the manufacturer’s instructions, with absorbance read at 562 nm using Infinite M Nano microplate reader (Tecan, Morgan Hill, CA, USA). Blank absorbance values (DMSO-BCA) were subtracted from the standard and sample absorbances to eliminate background signal. Sample concentrations were then determined by using the linear regression curve obtained from the standard dilutions. Loading was expressed as the mass of protein per total mass of microspheres (%, *w*/*w*).

### 4.4. In Vitro Release Testing

In vitro release studies were performed using an automated testing platform protocol. Briefly, lyophilized microspheres were resuspended in the chamber vials in 1 mL of release buffer (PBS, pH 7.4 at 37 °C) under static conditions. At predetermined intervals (every 24 h), the buffer was removed from the vials. The release buffer was collected for analysis and replaced with fresh release buffer after each sampling to maintain sink conditions.

Protein concentration in collected samples was determined using the BCA assay as described above. Release profiles were generated as a cumulative percentage of total protein released over time.

### 4.5. Statistical Analysis

Given a limited dataset (exploratory by design), we adopted a robust-first statistical framework. Analyses were conducted in Python 3.10 (Google Colaboratory; statsmodels, pingouin, scipy, pandas, numpy, matplotlib) and R 4.x (car, lmtest, boot). General linear models were fitted as Type-II ANCOVAs with heteroskedasticity-consistent (HC3) covariance estimators to correct standard errors under variance heterogeneity. Robustness of inferences was evaluated using resampling: Freedman–Lane permutation tests (10,000 iterations) and restricted wild bootstrap procedures (Rademacher multipliers, impose-the-null technique) for small samples. Partial η^2^ was reported as the effect size, and adjusted group differences were summarized using estimated marginal means (EMMs) with 95% confidence intervals. Complementary Spearman rank correlations were computed with false discovery rate (FDR) adjustment for multiple testing. Throughout, p denotes the unadjusted *p*-value, pHC3 the heteroskedasticity-consistent (HC3) Wald *p*-value, p_perm_ the Freedman–Lane permutation *p*-value, and q_FDR_ the Benjamini–Hochberg FDR-adjusted *p*-value; the symbol q is used exclusively for FDR-adjusted *p*-values and not as a studentized range statistic. Nonlinear regression of cumulative release profiles (t0–t7) to the Weibull function provided shape (β) and timescale (log τ), which were then analyzed by ANCOVA within the same framework. This workflow ensured that conclusions rested on converging evidence from model-based inference, robust resampling, and nonparametric correlation analysis, minimizing false positives under small-sample and imbalanced conditions. Where parametric and robust tests diverged, all three *p*-values—unadjusted, HC3, and permutation—are reported together, and effects that did not survive HC3 correction or permutation testing were treated as nominal and exploratory rather than confirmed.

To support these analyses, formulation- and release-related descriptors were first curated and transformed into consolidated parameters. Continuous variables (e.g., polymer viscosity, lactide content, drug concentration) were mean-centered and scaled where appropriate to reduce collinearity and aid interpretation of interaction terms. Categorical inputs (e.g., additive identity, end-cap chemistry) were grouped into mechanistic classes to ensure sufficient replication within levels and to capture shared functional properties. Burst and cumulative release measures at individual timepoints were reduced to derived parameters such as initial burst (t0), intermediate release (day-7), and Weibull β and log τ, summarizing trajectory shape and scale. These transformations allowed the raw experimental features to be expressed in a smaller set of interpretable covariates and outcome measures, harmonized across formulations. The final analytic dataset, therefore, reflected a balance between mechanistic granularity and statistical tractability, enabling consistent testing of factor effects across multiple complementary endpoints.

## 5. Conclusions

This study demonstrates that additive class and drug concentration in the dispersed phase are the formulation factors most strongly and reproducibly associated with antibody release from PLGA microspheres. Additive choice was the strongest correlate of burst release and kinetic pacing, while drug concentration modulated day-7 release. Lactide fraction exerted context-specific effects, and polymer intrinsic viscosity contributed negligibly. From a practical standpoint, these findings establish a design hierarchy: additives set the release framework, drug concentration fine-tunes early release, and lactide provides secondary modulation. Future studies with larger datasets and longer time spans are needed to confirm whether these early predictors also shape long-term release.

## Figures and Tables

**Table 1:** IgG loading as the percentage of the IgG in microspheres (*w*/*w*).

Formulation	Loading (%)	Formulation	Loading (%)	Formulation	Loading (%)
MA1	4.98	MA46	3.3	MA91	0
MA2	5.43	MA47	0.55	MA92	1.5
MA3	6.39	MA48	0.87	MA93	2.4
MA4	0.59	MA49	5.53	MA94	2.8
MA5	1.55	MA50	4.46	MA95	5
MA6	0	MA51	3.95	MA96	0.9
MA7	0.48	MA52	4.02	MA97	0.2
MA8	0	MA53	3.68	MA98	1.4
MA9	3.18	MA54	5.67	MA99	0
MA10	1.51	MA55	6.07	MA100	1
MA11	1.54	MA56	5.45	MA101	0.3
MA12	4.52	MA57	6.14	MA102	3.5
MA13	6.31	MA58	1.32	MA103	0
MA14	0.31	MA59	5.54	MA104	0
MA15	3.09	MA60	7.76	MA105	0
MA16	2.76	MA61	7.1	MA106	0
MA17	1.02	MA62	4.3	MA107	0
MA18	3.9	MA63	1.07	MA108	0.2
MA19	1.8	MA64	1.59	MA109	0
MA20	2.77	MA65	4.94	MA110	3.8
MA21	2.52	MA66	1.55	MA111	2
MA22	2.35	MA67	7.87	MA112	1.2
MA23	0.87	MA68	1.12	MA113	0
MA24	0.14	MA69	4.49	MA114	0
MA25	1.17	MA70	7.28	MA115	3.7
MA26	1.36	MA71	7.47	MA116	4.8
MA27	0.67	MA72	4.58	MA117	5.3
MA28	0.31	MA73	1.27	MA118	3
MA29	0.41	MA74	4.22	MA119	4.6
MA30	0.39	MA75	4.54	MA120	4.1
MA31	3.07	MA76	4.11	MA121	3.37
MA32	1.89	MA77	5.11	MA122	1.35
MA33	4.08	MA78	3.87	MA123	0.59
MA34	6.58	MA79	6.31	MA124	3.33
MA35	3.4	MA80	6.77	MA125	8.85
MA36	5.44	MA81	8.19	MA126	3.62
MA37	4.78	MA82	7.79	MA127	4.25
MA38	6.27	MA83	3.86	MA128	0
MA39	3.06	MA84	3.88	MA129	4.57
MA40	3.19	MA85	3.29		
MA41	0.93	MA86	3.51		
MA42	1.84	MA87	0.4		
MA43	1.97	MA88	2.31		
MA44	1.08	MA89	2.65		
MA45	1.54	MA90	3.45		

**Table 2:** Descriptive summary of outcomes by additive class. EMMs, 95% CI are shown for Drug Loading, Burst (t0), Day-7 release, and Weibull log τ, stratified by additive class (No-additive, Polymeric, Non-polymeric). ANCOVA F-statistics, HC3 *p*-values, permutation *p*-values, and partial η^2^ are reported. Robust, large effects were observed for burst and Weibull log τ, while drug loading and day-7 release showed weaker or non-robust associations.

Outcome	No-Additive (EMM, 95% CI)	Polymeric Additives (EMM, 95% CI)	Non-Polymeric Additives (EMM, 95% CI)	ANCOVA F (*p*)	HC3 (*p*)	Permutation (*p*)	Partial η^2^
Drug Loading (%)	3.46 (2.93–3.98)	2.82 (1.87–3.78)	1.70 (0.36–3.05)	2.84 (0.062)	0.096	0.466	0.041
Burst (t0, % release)	1.65 (−1.79, 5.09)	14.18 (8.75, 19.61)	24.50 (16.22, 32.79)	13.60 (1.2 × 10^−5^)	0.0018	0.0024	0.298
Day-7 Release (%)	18.18 (12.27, 24.08)	22.90 (13.59, 32.21)	40.57 (26.36, 54.78)	3.93 (0.0245)	0.209	0.248	0.109
Weibull log τ	−0.70 (−1.34, −0.06)	−3.24 (−4.35, −2.12)	−4.37 (−5.96, −2.78)	13.56 (1.4 × 10^−5^)	0.0008	0.0010	0.311

**Table 3:** Relative influence of predictors on outcomes. Additive class and drug concentration in the dispersed phase are the predictors most strongly and reproducibly associated with the outcomes, while lactide fraction shows context-specific effects, and polymer viscosity contributes minimally.

Predictor	Drug Loading	Burst (t0)	Day-7 Release
Additive class	Weak (η^2^ = 0.041, not robust)	Large (η^2^ = 0.298, robust)	Nominal (η^2^ = 0.109, not robust)
Drug conc. in DP	Negative trend (ρ ≈ –0.29)	Negligible	Large effect (η^2^ = 0.152, robust)
Lactide fraction	Negligible	Trend only (η^2^ ≈ 0.051, not robust)	Small

**Table 4:** Summary of evidential support across statistical methods. For each outcome, the parametric ANCOVA, HC3-robust, and Freedman–Lane permutation *p*-values are shown together with the effect size (partial η^2^) and an overall evidence class: robust (concordant across all three methods), nominal (significant under the parametric test only and not surviving HC3 correction or permutation), or exploratory (supported only by correlation analysis).

Outcome	ANCOVA *p*	HC3 *p*	Permutation *p*	Partial η^2^	Evidence Class
Burst (t0)	1.2 × 10^−5^	0.0018	0.0024	0.298	Robust (concordant across all three methods)
Weibull log τ	1.4 × 10^−5^	0.0008	0.0010	0.311	Robust (concordant across all three methods)
Day-7 release	0.0245	0.209	0.248	0.109	Nominal (parametric only; not robust)
Drug loading	0.062	0.096	0.466	0.041	Exploratory (correlation-level only)

## Data Availability

The original contributions presented in this study are included in the article/Supplementary Material. Further inquiries can be directed to the corresponding author.

## Supplementary Materials

The following supporting information can be downloaded at: https://www.mdpi.com/article/10.3390/ph19081203/s1, Table S1. List of Materials. Table S2. List of Formulations. Each formulation shows the parameter variable used in the formulation building. Table S3. List of Formulation groups. Formulations are grouped using parameter variables.

## Abbreviations

The following abbreviations are used in this manuscript:

ANCOVA	Analysis of covariance
BCA	Bicinchoninic acid
CP	Continuous phase
DP	Dispersed phase
LAI	Long-acting injectables
pHC3	Heteroskedasticity-consistent (HC3) Wald *p*-value
PLA	Polylactic acid
PLGA	Poly(lactic-co-glycolic) acid
p_perm_	Freedman–Lane permutation *p*-value
q_FDR_	False discovery rate (FDR)-adjusted *p*-value (Benjamini–Hochberg method)
