# Supplementary material for: Exploration of Development Parameters for IgG Microspheres Using an Automated Platform for High-Throughput Formulation Screening
Source: Pharmaceuticals (Basel). 2026 Aug 1;19(8):1203. doi: 10.3390/ph19081203 (PMC13517076; doi:10.3390/ph19081203)
Supplement: Supplementary file 1 [file pharmaceuticals-19-01203-s001.zip › pharmaceuticals-4329642-supplementary.pdf]

Supplementary Materials

Table S1. List of Materials.

**Polymer**	**Supplier**
Expansorb 10-P026 (50:50 PLGA, i.v. 0.71-0.86)	Seqens
Expansorb 10-P037 (50:50 PLGA, i.v. 0.55-0.70)	Seqens
Expansorb 10-P046 (75:25 PLGA, i.v. 0.5-0.7)	Seqens
Expansorb 10-P048 (75:25 PLGA, i.v. 0.7-0.9)	Seqens
Expansorb 10-P045 (PLA, i.v. 0.33-0.58)	Seqens
Expansorb 10-P047 (PLA, i.v. 0.59-0.84)	Seqens
**Excipient**	**Supplier**
Dichloromethane	Thermo Scientific
Poly(vinyl alcohol), 88% hydrolyzed, MW 85-120k	Thermo Scientific
Alginic acid	Sigma Aldrich
Dextran sulfate sodium salt, MW 500k	Sigma Aldrich
Distearoyl-sn-glycero-3-phosphocholine	TCI
Heparin sodium salt	Sigma Aldrich
Poly(allylamine hydrochloride)	Alfa Aesar
Arginine, 98+%	Thermo Scientific
Ammonium Sulfate	Sigma Aldrich
Polyethyleneimine	Thermo Scientific
Poly-L-lysine hydrobromide	Sigma Aldrich
Sodium hyaluronate, 95%	Thermo scientific
Polysorbate 80	Spectrum
Polyacrylamide	Thermo Scientific
Chitosan (from shrimp shells, practical grade)	Sigma Aldrich

Table S2. List of Formulations. Each formulation shows the parameter variable used in the formulation building.

**Formulation**	**IgG in DP (mg/ml)**	**Primary polymer**	**Secondary polymer**	**Polymer ratio**	**Polymer in DP (%)**	**Additive**
MA1	2	Expansorb 10-P047			6.7%	
MA2	2	Expansorb 10-P048			6.7%	
MA3	2	Expansorb 10-P026			6.7%	
MA4	10	Expansorb 10-P026			6.7%	
MA5	1.25	Expansorb 10-P037	Expansorb 10-P047	3:1	3.3%	

MA6	1.25	Expansorb 10-P026			3.3%	
MA7	3.75	Expansorb 10-P037			6.7%	
MA8	3.75	Expansorb 10-P037	Expansorb 10-P047	3:1	6.7%	
MA9	3.75	Expansorb 10-P037	Expansorb 10-P047	1:1	6.7%	
MA10	3.75	Expansorb 10-P037	Expansorb 10-P047	1:3	6.7%	
MA11	3.75	Expansorb 10-P026	Expansorb 10-P045	3:1	6.7%	
MA12	3.75	Expansorb 10-P026	Expansorb 10-P045	1:1	6.7%	
MA13	3.75	Expansorb 10-P026	Expansorb 10-P045	1:3	6.7%	
MA14	3.75	Expansorb 10-P047	Expansorb 10-P037	1:3	3.3%	
MA15	3.75	Expansorb 10-P037			3.3%	
MA16	3.75	Expansorb 10-P048	Expansorb 10-P046	3:1	3.3%	
MA17	3.75	Expansorb 10-P048	Expansorb 10-P046	1:1	3.3%	
MA18	3.75	Expansorb 10-P048	Expansorb 10-P046	1:3	3.3%	
MA19	3.75	Expansorb 10-P047	Expansorb 10-P037	1:1	6.7%	
MA20	3.75	Expansorb 10-P048			6.7%	
MA21	3.75	Expansorb 10-P048	Expansorb 10-P046	3:1	6.7%	
MA22	3.75	Expansorb 10-P046			6.7%	
MA23	3.75	Expansorb 10-P047			3.3%	
MA24	3.75	Expansorb 10-P047	Expansorb 10-P045	3:1	3.3%	
MA25	3.75	Expansorb 10-P047			6.7%	
MA26	3.75	Expansorb 10-P037			6.7%	
MA27	3.75	Expansorb 10-P047			6.7%	

MA28	3.75	Expansorb 10-P047	Expansorb 10-P037	3:1	6.7%	
MA29	3.75	Expansorb 10-P047	Expansorb 10-P037	3:1	6.7%	
MA30	3.75	Expansorb 10-P037			6.7%	
MA31	1.25	Expansorb 10-P037			3.3%	
MA32	1.25	Expansorb 10-P037	Expansorb 10-P047	3:1	3.3%	
MA33	1.25	Expansorb 10-P037	Expansorb 10-P047	1:1	3.3%	
MA34	1.25	Expansorb 10-P037	Expansorb 10-P047	1:3	3.3%	
MA35	1.25	Expansorb 10-P047			3.3%	
MA36	3.75	Expansorb 10-P037			6.7%	
MA37	3.75	Expansorb 10-P037	Expansorb 10-P047	3:1	6.7%	
MA38	3.75	Expansorb 10-P037	Expansorb 10-P047	1:1	6.7%	
MA39	3.75	Expansorb 10-P037	Expansorb 10-P047	1:3	6.7%	
MA40	3.75	Expansorb 10-P037			6.7%	
MA41	3.75	Expansorb 10-P047			3.3%	
MA42	3.75	Expansorb 10-P047	Expansorb 10-P037	3:1	3.3%	
MA43	3.75	Expansorb 10-P047	Expansorb 10-P037	1:1	3.3%	
MA44	3.75	Expansorb 10-P037			3.3%	
MA45	3.75	Expansorb 10-P048			3.3%	
MA46	3.75	Expansorb 10-P048	Expansorb 10-P046	1:1	3.3%	
MA47	3.75	Expansorb 10-P048	Expansorb 10-P046	1:3	3.3%	
MA48	3.75	Expansorb 10-P046			3.3%	
MA49	3.75	Expansorb 10-P047			6.7%	

MA50	3.75	Expansorb 10-P047	Expansorb 10-P037	3:1	6.7%	
MA51	3.75	Expansorb 10-P047	Expansorb 10-P037	1:1	6.7%	
MA52	3.75	Expansorb 10-P047	Expansorb 10-P037	1:3	6.7%	
MA53	3.75	Expansorb 10-P037			6.7%	
MA54	3.75	Expansorb 10-P048	Expansorb 10-P046	3:1	6.7%	
MA55	3.75	Expansorb 10-P048	Expansorb 10-P046	1:1	6.7%	
MA56	3.75	Expansorb 10-P048	Expansorb 10-P046	1:3	6.7%	
MA57	3.75	Expansorb 10-P047			3.3%	
MA58	3.75	Expansorb 10-P047	Expansorb 10-P045	3:1	3.3%	
MA59	3.75	Expansorb 10-P047	Expansorb 10-P045	1:1	3.3%	
MA60	3.75	Expansorb 10-P047	Expansorb 10-P045	1:3	3.3%	
MA61	3.75	Expansorb 10-P045			3.3%	
MA62	3.75	Expansorb 10-P047			6.7%	
MA63	3.75	Expansorb 10-P047	Expansorb 10-P045	3:1	6.7%	
MA64	3.75	Expansorb 10-P047	Expansorb 10-P045	1:1	6.7%	
MA65	3.75	Expansorb 10-P047	Expansorb 10-P045	1:3	6.7%	
MA66	3.75	Expansorb 10-P045			6.7%	
MA67	3.75	Expansorb 10-P047			3.3%	
MA68	3.75	Expansorb 10-P047	Expansorb 10-P037	3:1	3.3%	
MA69	3.75	Expansorb 10-P047	Expansorb 10-P037	1:1	3.3%	
MA70	3.75	Expansorb 10-P047	Expansorb 10-P037	1:3	3.3%	
MA71	3.75	Expansorb 10-P037			3.3%	

MA72	3.75	Expansorb 10-P047			6.7%	
MA73	3.75	Expansorb 10-P047	Expansorb 10-P037	3:1	6.7%	
MA74	3.75	Expansorb 10-P047	Expansorb 10-P037	1:1	6.7%	
MA75	3.75	Expansorb 10-P047	Expansorb 10-P037	1:3	6.7%	
MA76	3.75	Expansorb 10-P037			6.7%	
MA77	3.75	Expansorb 10-P047			6.7%	
MA78	3.75	Expansorb 10-P037	Expansorb 10-P047	1:3	6.7%	
MA79	3.75	Expansorb 10-P047			3.3%	
MA80	3.75	Expansorb 10-P047	Expansorb 10-P045	3:1	3.3%	
MA81	3.75	Expansorb 10-P047	Expansorb 10-P045	1:1	3.3%	
MA82	3.75	Expansorb 10-P045			3.3%	
MA83	3.75	Expansorb 10-P047	Expansorb 10-P045	3:1	6.7%	
MA84	3.75	Expansorb 10-P047	Expansorb 10-P045	1:1	6.7%	
MA85	3.75	Expansorb 10-P047	Expansorb 10-P045	1:3	6.7%	
MA86	3.75	Expansorb 10-P046			6.7%	
MA87	3.75	Expansorb 10-P037	Expansorb 10-P048	1:1	6.7%	
MA88	3.75	Expansorb 10-P037	Expansorb 10-P048	1:1	3.3%	
MA89	3.75	Expansorb 10-P037	Expansorb 10-P048	1:1	3.3%	
MA90	3.75	Expansorb 10-P037	Expansorb 10-P048	1:3	3.3%	
MA91	3.75	Expansorb 10-P037			3.3%	1% Alginic Acid
MA92	3.75	Expansorb 10-P046			3.3%	1% Alginic Acid
MA93	3.75	Expansorb 10-P047			3.3%	1% Alginic Acid

MA94	3.75	Expansorb 10-P037			3.3%	1% Dextran Sulfate
MA95	3.75	Expansorb 10-P046			3.3%	1% Dextran Sulfate
MA96	3.75	Expansorb 10-P046			3.3%	1% Distearoyl-sn-glycero-phosphocholin in DCM
MA97	3.75	Expansorb 10-P046			3.3%	1% Heparin
MA98	3.75	Expansorb 10-P046			3.3%	1% Polyallylamine HCl
MA99	3.75	Expansorb 10-P047			3.3%	1% Dextran Sulfate
MA100	3.75	Expansorb 10-P047			3.3%	1% Distearoyl-sn-glycero-phosphocholin in DCM
MA101	3.75	Expansorb 10-P047			3.3%	1% Heparin
MA102	3.75	Expansorb 10-P037			3.3%	1% Heparin
MA103	3.75	Expansorb 10-P037			3.3%	1% Distearoyl-sn-glycero-phosphocholin in DCM
MA104	3.75	Expansorb 10-P037			3.3%	1% Polyallylamine HCl
MA105	3.75	Expansorb 10-P047			3.3%	1% Polyallylamine HCl
MA106	3.75	Expansorb 10-P047			3.3%	1% Arginine in Water
MA107	3.75	Expansorb 10-P047			3.3%	1% Ammonium Sulfate
MA108	3.75	Expansorb 10-P047			3.3%	0.5% Polyethyleneimine
MA109	3.75	Expansorb 10-P037			3.3%	1% Arginine
MA110	3.75	Expansorb 10-P037			3.3%	1% Ammonium Sulfate
MA111	3.75	Expansorb 10-P037			3.3%	0.5% Polyethyleneimine
MA112	3.75	Expansorb 10-P046			3.3%	1% Arginine
MA113	3.75	Expansorb 10-P046			3.3%	1% Ammonium Sulfate
MA114	3.75	Expansorb 10-P046			3.3%	0.5% Polyethyleneimine
MA115	3.75	Expansorb 10-P037			3.3%	1% Poly-L-Lysine

MA116	3.75	Expansorb 10-P037			3.3%	1% Chitosan
MA117	3.75	Expansorb 10-P037			3.3%	0.5% Na-Hyaluronate
MA118	3.75	Expansorb 10-P037			3.3%	1% Polysorbate 80
MA119	3.75	Expansorb 10-P047			3.3%	0.5% Na-Hyaluronate
MA120	3.75	Expansorb 10-P047			3.3%	1% Polysorbate 80
MA121	3.75	Expansorb 10-P047			3.3%	1% Poly-L-Lysine
MA122	3.75	Expansorb 10-P046			3.3%	1% Poly-L-Lysine
MA123	3.75	Expansorb 10-P046			3.3%	1% Polysorbate 80
MA124	3.75	Expansorb 10-P046			3.3%	0.5% Na-Hyaluronate
MA125	3.75	Expansorb 10-P046			3.3%	1% Polyacrylamide
MA126	3.75	Expansorb 10-P046			3.3%	1% Chitosan
MA127	3.75	Expansorb 10-P047			3.3%	1% Chitosan
MA128	3.75	Expansorb 10-P047			3.3%	1% Polyacrylamide
MA129	3.75	Expansorb 10-P037			3.3%	1% Polyacrylamide

Table S3. List of Formulation groups. Formulations are grouped using parameter variables.

Parameter	Variable	No. of formulations	Formulations
**IgG in DP (mg/ml)**	10.0 mg/mL	1	MA4
	2.0 mg/mL	3	MA1, MA2, MA3
	1.25 mg/mL	7	MA31, MA32, MA33, MA34, MA35, MA5, MA6

	3.75 mg/mL	118	MA10, MA100, MA101, MA102, MA103, MA104, MA105, MA106, MA107, MA108, MA109, MA11, MA110, MA111, MA112, MA113, MA114, MA115, MA116, MA117, MA118, MA119, MA12, MA120, MA121, MA122, MA123, MA124, MA125, MA126, MA127, MA128, MA129, MA13, MA14, MA15, MA16, MA17, MA18, MA19, MA20, MA21, MA22, MA23, MA24, MA25, MA26, MA27, MA28, MA29, MA30, MA36, MA37, MA38, MA39, MA40, MA41, MA42, MA43, MA44, MA45, MA46, MA47, MA48, MA49, MA50, MA51, MA52, MA53, MA54, MA55, MA56, MA57, MA58, MA59, MA60, MA61, MA62, MA63, MA64, MA65, MA66, MA67, MA68, MA69, MA7, MA70, MA71, MA72, MA73, MA74, MA75, MA76, MA77, MA78, MA79, MA8, MA80, MA81, MA82, MA83, MA84, MA85, MA86, MA87, MA88, MA89, MA9, MA90, MA91, MA92, MA93, MA94, MA95, MA96, MA97, MA98, MA99
**Primary polymer**	Expansorb 10-P045	3	MA61, MA66, MA82
	Expansorb 10-P026	6	MA11, MA12, MA13, MA3, MA4, MA6
	Expansorb 10-P048	12	MA16, MA17, MA18, MA2, MA20, MA21, MA45, MA46, MA47, MA54, MA55, MA56
	Expansorb 10-P046	16	MA112, MA113, MA114, MA122, MA123, MA124, MA125, MA126, MA22, MA48, MA86, MA92, MA95, MA96, MA97, MA98
	Expansorb 10-P037	39	MA10, MA102, MA103, MA104, MA109, MA110, MA111, MA115, MA116, MA117, MA118, MA129, MA15, MA26, MA30, MA31, MA32, MA33, MA34, MA36, MA37, MA38, MA39, MA40, MA44, MA5, MA53, MA7, MA71, MA76, MA78, MA8, MA87, MA88, MA89, MA9, MA90, MA91, MA94
	Expansorb 10-P047	53	MA1, MA100, MA101, MA105, MA106, MA107, MA108, MA119, MA120, MA121, MA127, MA128, MA14, MA19, MA23, MA24, MA25, MA27, MA28, MA29, MA35, MA41, MA42, MA43, MA49, MA50, MA51, MA52, MA57, MA58, MA59, MA60, MA62, MA63,

			MA64, MA65, MA67, MA68, MA69, MA70, MA72, MA73, MA74, MA75, MA77, MA79, MA80, MA81, MA83, MA84, MA85, MA93, MA99
**Secondary polymer**	Expansorb 10-P048	4	MA87, MA88, MA89, MA90
	Expansorb 10-P046	9	MA16, MA17, MA18, MA21, MA46, MA47, MA54, MA55, MA56
	Expansorb 10-P047	11	MA10, MA32, MA33, MA34, MA37, MA38, MA39, MA5, MA78, MA8, MA9
	Expansorb 10-P037	15	MA14, MA19, MA28, MA29, MA42, MA43, MA50, MA51, MA52, MA68, MA69, MA70, MA73, MA74, MA75
	Expansorb 10-P045	15	MA11, MA12, MA13, MA24, MA58, MA59, MA60, MA63, MA64, MA65, MA80, MA81, MA83, MA84, MA85
	None	75	MA1, MA100, MA101, MA102, MA103, MA104, MA105, MA106, MA107, MA108, MA109, MA110, MA111, MA112, MA113, MA114, MA115, MA116, MA117, MA118, MA119, MA120, MA121, MA122, MA123, MA124, MA125, MA126, MA127, MA128, MA129, MA15, MA2, MA20, MA22, MA23, MA25, MA26, MA27, MA3, MA30, MA31, MA35, MA36, MA4, MA40, MA41, MA44, MA45, MA48, MA49, MA53, MA57, MA6, MA61, MA62, MA66, MA67, MA7, MA71, MA72, MA76, MA77, MA79, MA82, MA86, MA91, MA92, MA93, MA94, MA95, MA96, MA97, MA98, MA99
**Polymer ratio**	1:3	17	MA10, MA13, MA14, MA18, MA29, MA34, MA39, MA47, MA52, MA56, MA60, MA65, MA70, MA75, MA78, MA85, MA90
	3:1	18	MA11, MA16, MA21, MA24, MA28, MA32, MA37, MA42, MA5, MA50, MA54, MA58, MA63, MA68, MA73, MA8, MA80, MA83
	1:1	19	MA12, MA17, MA19, MA33, MA38, MA43, MA46, MA51, MA55, MA59, MA64, MA69, MA74, MA81, MA84, MA87, MA88, MA89, MA9

	None	75	MA1, MA44, MA100, MA101, MA102, MA103, MA104, MA105, MA106, MA107, MA108, MA109, MA110, MA111, MA112, MA113, MA114, MA115, MA116, MA117, MA118, MA119, MA120, MA121, MA122, MA123, MA124, MA125, MA126, MA127, MA128, MA129, MA15, MA2, MA20, MA22, MA23, MA25, MA26, MA27, MA3, MA30, MA31, MA35, MA36, MA4, MA40, MA41, MA45, MA48, MA49, MA53, MA57, MA6, MA61, MA62, MA66, MA67, MA7, MA71, MA72, MA76, MA77, MA79, MA82, MA86, MA91, MA92, MA93, MA94, MA95, MA96, MA97, MA98, MA99
**Polymer concentration in DP (%)**	6.70%	51	MA1, MA10, MA11, MA12, MA13, MA19, MA2, MA20, MA21, MA22, MA25, MA26, MA27, MA28, MA29, MA3, MA30, MA36, MA37, MA38, MA39, MA4, MA40, MA49, MA50, MA51, MA52, MA53, MA54, MA55, MA56, MA62, MA63, MA64, MA65, MA66, MA7, MA72, MA73, MA74, MA75, MA76, MA77, MA78, MA8, MA83, MA84, MA85, MA86, MA87, MA9
	3.30%	78	MA100, MA101, MA102, MA103, MA104, MA105, MA106, MA107, MA108, MA109, MA110, MA111, MA112, MA113, MA114, MA115, MA116, MA117, MA118, MA119, MA120, MA121, MA122, MA123, MA124, MA125, MA126, MA127, MA128, MA129, MA14, MA15, MA16, MA17, MA18, MA23, MA24, MA31, MA32, MA33, MA34, MA35, MA41, MA42, MA43, MA44, MA45, MA46, MA47, MA48, MA5, MA57, MA58, MA59, MA6, MA60, MA61, MA67, MA68, MA69, MA70, MA71, MA79, MA80, MA81, MA82, MA88, MA89, MA90, MA91, MA92, MA93, MA94, MA95, MA96, MA97, MA98, MA99
**Additive**	0.5% Na-Hyaluronate	3	MA117, MA119, MA124
	0.5% Polyethyleneimine	3	MA108, MA111, MA114
	1% Alginic Acid	3	MA91, MA92, MA93

	1% Ammonium Sulfate	3	MA107, MA110, MA113
	1% Arginine	3	MA106, MA109, MA112
	1% Chitosan	3	MA116, MA126, MA127
	1% Dextran Sulfate	3	MA94, MA95, MA99
	1% Distearoyl-sn-glycero-phosphocholin in DCM	3	MA100, MA103, MA96
	1% Heparin	3	MA101, MA102, MA97
	1% Poly-L-Lysine	3	MA115, MA121, MA122
	1% Poly-acrylamide	3	MA125, MA128, MA129
	1% Polyallylamine	3	MA104, MA105, MA98
	1% Polysorbate 80	3	MA118, MA120, MA123
	None	90	MA1, MA10, MA11, MA12, MA13, MA14, MA15, MA16, MA17, MA18, MA19, MA2, MA20, MA21, MA22, MA23, MA24, MA25, MA26, MA27, MA28, MA29, MA3, MA30, MA31, MA32, MA33, MA34, MA35, MA36, MA37, MA38, MA39, MA4, MA40, MA41, MA42, MA43, MA44, MA45, MA46, MA47, MA48, MA49, MA5, MA50, MA51, MA52, MA53, MA54, MA55, MA56, MA57, MA58, MA59, MA6, MA60, MA61, MA62, MA63, MA64, MA65, MA66, MA67, MA68, MA69, MA7, MA70, MA71, MA72, MA73, MA74, MA75, MA76, MA77, MA78, MA79, MA8, MA80, MA81, MA82, MA83, MA84, MA85, MA86, MA87, MA88, MA89, MA9, MA90
